# Supplementary material for: Expectation-driven gain modulation of preparatory spatial attention
Source: eLife. 2026 Nov 6;15:RP110315. doi: 10.7554/eLife.110315 (PMC13743075; doi:10.7554/eLife.110315)
Supplement: Peer review history

# Editor report: eLife Assessment

**DOI:** 10.7554/eLife.110315.3.sa0

**Authors:** Redmond G O'Connell

**Affiliations:** Trinity College Dublin Ireland

**Keywords:** Solid, Valuable

This study provides **valuable** evidence regarding our expectations about task difficulty and how this might influence proactive attention. The findings suggest that anticipated demands enhance the strength of attentional selection at cued locations. The evidence is **solid** but not definitive, as the conclusions rely on the absence of changes in spatial breadth. Nevertheless, the manuscript puts forth an informative experiment with a well written and thoughtful discussion of the results.

# Referee report: Reviewer #1 (Public review)

**DOI:** 10.7554/eLife.110315.3.sa1

Summary:

The authors attempt to use a combination of behavioural and EEG analyses in order to investigate whether expectation of task difficulty influences spatial focus narrowing in the context of a spatially cued task, alongside an expected attention-related amplitude effect. This distinguishes the experiment from previous tasks which looked at this potential spatial narrowing in the context of more non-cued diffuse attention tasks. The authors present 2 major findings.

(1) Behaviourally, they analysed the effects of cue validity and difficulty expectation on response accuracy and found that participants displayed an effect of difficulty expectation in validly cued trials, showing relatively enhanced behaviour to Hard Expectation trials, but no effect of expectation in invalidly cued trials.

(2) Inverted encoding modelling on broadband EEG showed greater pre-target attentional processing in the Hard Expectation blocks. They go on to show that this enhancement comes in the form of greater amplitude of the Channel Tuning Functions (CTFs) approximately 300 to 400ms post-cue, in the absence of any spatial tuning specificity enhancement (as would be evident in a difference in CTF fit width). Together these results provide valuable findings for those investigating the separable effects of expectation and attention on target detection in visual search.

Strengths:

(1) This is a very solidly performed experiment and analysis, with different streams of evidence convincingly pointing in the same direction, i.e. a gain effect of Expectation in the absence of a spatial tuning effect.

(2) EEG is competently analysed and interpreted, and the paper is well written, and simple in its motivation.

(3) The authors report appropriately on the results in the Discussion, without overreaching.

Comments on revised version:

The authors have addressed all of my comments. Very interesting work, thank you!

# Referee report: Reviewer #2 (Public review)

**DOI:** 10.7554/eLife.110315.3.sa2

Summary:

The authors set out to determine whether people can adjust how narrowly or broadly they focus attention in advance based on expectations about how difficult an upcoming visual task will be. Specifically, they aimed to test whether expecting a more demanding search leads to a narrower focus of attention or instead strengthens attention at the relevant location without changing its spatial extent.

Strengths:

The study addresses a timely and interesting question about how expectations influence the preparation of attention before a task begins. The experimental design is well suited to isolating anticipatory effects by manipulating expectations about task difficulty independently of moment-to-moment stimulus information. The manuscript is clearly written, and the methods are described in sufficient detail to support transparency and reproducibility.

Comments on revised version.

During the review process the authors addressed my previous concerns. The revisions have improved the clarity of the analyses and the interpretation of the results, and I have no further substantive comments.

# Author comment: Author response

**DOI:** 10.7554/eLife.110315.3.sa3

**Authors:** Dirk van Moorselaar, Stefan van der Stigchel

**Affiliations:** Utrecht University Amsterdam Netherlands; Utrecht University Utrecht Nepal

The following is the authors’ response to the original reviews.

In the revised manuscript, we have implemented several substantive changes. Most notably, we have revised the statistical reporting throughout to use Wald z statistics and GLMM-based contrasts, replacing the previously reported F statistics and figure caption t-tests. We have also expanded the Discussion to more explicitly acknowledge interpretational caveats regarding the null tuning width result and to address the alternative explanation of general alertness or motivational changes. Throughout the manuscript, we have revised our language to ensure that our conclusions are appropriately calibrated to the data.

**Reviewer #1 (Public review):**

Summary:

The authors attempt to use a combination of behavioural and EEG analyses in order to investigate whether expectation of task difficulty influences spatial focus narrowing in the context of a spatially cued task, alongside an expected attention-related amplitude effect. This distinguishes the experiment from previous tasks, which looked at this potential spatial narrowing in the context of more non-cued diffuse attention tasks. The authors present two major findings:

(1) Behaviourally, they analysed the effects of cue validity and difficulty expectation on response accuracy, and found that participants displayed an effect of difficulty expectation in validly cued trials, showing relatively enhanced behaviour to Hard Expectation trials, but no effect of expectation in invalidly cued trials.

(2) Inverted encoding modelling on broadband EEG showed greater pre-target attentional processing in the Hard Expectation blocks. They go on to show that this enhancement comes in the form of greater amplitude of the Channel Tuning Functions (CTFs) approximately 300 to 400ms post-cue, in the absence of any spatial tuning specificity enhancement (as would be evident in a difference in CTF fit width).

Together, these results provide valuable findings for those investigating the separable effects of expectation and attention on target detection in visual search.

Strengths:

(1) This is a very solidly performed experiment and analysis, with different streams of evidence convincingly pointing in the same direction, i.e. a gain effect of Expectation in the absence of a spatial tuning effect.

(2) EEG is competently analysed and interpreted, and the paper is well written and simple in its motivation.

(3) The authors report appropriately on the results in the Discussion, without overreaching.

Weaknesses:

I mainly have a few minor issues for the authors to clarify, which I will leave to Recommendations. However, a few analyses need further work:

We thank Reviewer 1 for the overall positive evaluation of our work and for the constructive and detailed feedback. The reviewer highlighted several strengths of the study, including the convergent evidence across behavioral and neural measures, the competent EEG analysis, and the appropriateness of the Discussion. In response to the specific recommendations, we have: clarified the type of EEG analysis in the Abstract; revised the description of the Serences et al. (2004) finding in the Introduction; added a Figure 1 reference in the relevant paragraph; clarified the logic of the planned comparisons; corrected and updated Figure 2 and its caption; added clarifying information about the EEG analysis in the Results; corrected the ambiguous reference to stimulus onset; clarified the status of edge-marked participants in Figure 4a; and added caveats and clarifications regarding the decoding analysis. We also address the two analytical concerns raised under Weaknesses below.

(1) The GLMM method used has very large degrees of freedom (pages 6 and 7) of 34542. I assume this is the number of trials minus the number of parameters? This would imply that random slopes were not modelled in the analyses. However, looking at the Methods, it is reported that they were modelled. The authors should clarify exactly what was done here and why, including the LMM model.

We thank the reviewer for raising this point. The previously reported denominator degrees of freedom (e.g., 34,542) reflected the number of trial-level observations used in the model and arose from reporting Type III Wald F-tests. We agree that this reporting format may have been misleading in the context of generalized linear mixed-effects models (GLMMs), where inference does not rely on classical denominator degrees of freedom in the same way as traditional ANOVA.

To improve clarity, we have revised the manuscript to report fixed effects using Wald z statistics derived from the model summary, which is the standard approach for binomial GLMMs implemented in

The revised reporting yields the same overall pattern of results, with the key planned comparison remaining significant.

(2) Figure 4 shows an "example CTF fit". Why only one? You could put transparent lines in the background for each individual fit, followed by the grand average, or show each fit in the supplementary section?

We thank the reviewer for this suggestion. We would like to clarify that Figure 4 does not show an example single-subject CTF fit; it shows the CTF fit to the group-averaged data, i.e., the grand average across participants. The purpose of the figure is to illustrate the group-level tuning function. This is now clarified in the updated Figure caption.

To convey individual differences, Figure 4a already presents the parameter estimates for each participant (width, amplitude, and baseline) as separate points, providing a clear view of variability across participants. We considered including individual CTF fits in the background, but this would make the figure crowded without adding interpretive value, since the individual parameters are already visualized.

We could, if the reviewers prefer, include the individual fits in the Supplementary Material; however, we believe that the current presentation conveys both the group average and participant-level variation clearly.

**Reviewer #1 (Recommendations for the authors):**

(3) Specify what type of EEG results are found in the Abstract. It is broadband, but one might expect, e.g. Alpha analyses.

We thank the reviewer for this suggestion. We have added "broadband" to the Abstract when describing the EEG analysis approach, clarifying that the inverted encoding model was applied to broadband EEG data rather than a specific frequency band (e.g., alpha).

“We applied inverted encoding models to broadband EEG data to reconstruct spatial channel tuning functions, enabling precise characterization of both the locus and breadth of attentional deployment.”

(4) In the Intro, please clarify the Serences finding that they found enhanced activity at expected distractor locations. The interpretation is that this reflects preparatory tagging of where distractors will appear, possibly to facilitate their suppression once they arrive, rather than enhancement in the service of processing those locations. It is confusing as it is currently worded.

We thank the reviewer for flagging this. We have revised the description of the Serences et al. (2004) finding to clarify that the enhanced activity at expected distractor locations is interpreted as preparatory tagging in service of subsequent suppression, rather than signal enhancement facilitating processing at those locations. The revised sentence now makes this interpretive distinction explicit.

“Complementing these findings, Serences et al. (2004) used fMRI to show that preparatory attention when expecting high distractor interference selectively enhanced activity in early visual cortex at retinotopic locations corresponding to the expected distractor positions, an effect interpreted as preparatory tagging of distractor locations to facilitate their subsequent suppression.”

(5) Page 6: refer to Figure 1 in the relevant paragraph.

We thank the reviewer for this suggestion. We have added a reference to Figure 1 in the relevant paragraph to help orient the reader.

(6) Page 7: I find the interaction confusing. The authors say there is an interaction of Expectation and Cue Validity, such that there is a larger cueing benefit when dense displays were expected. However, this leads one to expect planned comparisons between Valid vs Invalid for Easy then Hard expectations. However, that's not what is done, actually comparing Easy vs Hard for Valid then Invalid trials.

We thank the reviewer for highlighting this potential source of confusion. We have clarified in the manuscript that the planned comparisons examined the effect of Expectation separately within valid and invalid trials, rather than comparing cueing effects (valid vs. invalid) within each Expectation level. This analytic approach was chosen to directly test our hypothesis regarding expectation-related modulation of performance at attended versus unattended locations. We hope this clarification makes the logic of the comparisons more transparent.

“To identify the locus of this interaction, we examined the effect of Expectation separately within valid and invalid trials, allowing us to test whether expectations exerted their effects at both cued and uncued locations, or selectively at either cued or uncued locations.”

(7) Page 7: Issue with asterisk in Figure 2. Text says it is not significant. Also, can you make the transparent grey lines more visible? Also, the inner plot shows two sets of lines, apparently easy and hard display results. Needs to be denoted.

We thank the reviewer for these observations. We have made the following changes: (1) The pairwise comparisons reported in the figure caption have been replaced with contrasts derived from the GLMM using estimated marginal means, consistent with the statistical approach used throughout the manuscript. (2) We have corrected the asterisk annotation in Figure 2, which was incorrectly placed on a non-significant comparison. (3) We have increased the visibility of the transparent grey lines in the figure. (4) We have revised the figure such that it is visually clear that the legend from the main plot applies to the inset plot as well.

(8) Page 8: Really need some info on the EEG analysis.

We thank the reviewer for this suggestion. We have added a sentence to the Results section briefly explaining that CTF slope reflects the overall strength of spatially selective neural activity at the attended location, with steeper slopes indicating stronger spatial selectivity, before directing readers to the Methods for full technical details. We hope this provides sufficient context for readers less familiar with the IEM approach without overloading the Results with methodological detail.

(9) Page 8: 100ms after stimulus onset = target or cue? From Figure 4, it seems to be a cue, but this really needs to be clarified.

We thank the reviewer for catching this ambiguity. We have replaced "stimulus onset" with "cue onset" throughout the results section to make clear that the time course is locked to cue presentation rather than target onset.

(10) Page 10: Figure 4a, are edge-marked participants outliers? Were they included in analyses?

We thank the reviewer for this observation. The edge-marked data points in Figure 4a reflect the default matplotlib boxplot visualization, which flags points beyond 1.5 × IQR, and do not represent a formal outlier exclusion criterion. We have added a brief clarification to this effect in the figure caption. All participants were retained in the primary analyses. To confirm that these participants did not unduly influence the results, we conducted a sensitivity analysis excluding them. Notably, the flagged participant showed a pattern in the opposite direction to the group, and excluding this individual yielded a stronger and more consistent effect, suggesting that our primary analysis with all participants included represents a conservative estimate.

(11) Page 11: Can't infer the same mechanism from the lack of decoding ability; it could be a signal-to-noise issue. However, one interesting question. How is it that the Encoding analysis worked out, but the Decoding analysis did not?

We thank the reviewer for raising both points. We agree that chance decoding could in principle reflect limited sensitivity rather than a true null effect, and we have added a caveat acknowledging this in the manuscript. We have also added a clarifying sentence explaining the complementary nature of the IEM and decoding analyses: the IEM captures the strength of spatial tuning within each condition, whereas decoding tests whether spatial patterns differ between conditions. Amplitude modulation of a shared spatial pattern would not necessarily produce discriminable multivariate patterns, which explains why the IEM detected amplitude differences while decoding remained at chance. We hope this resolves the apparent paradox.

**Reviewer #2 (Public review):**

Summary:

The authors set out to determine whether people can adjust how narrowly or broadly they focus attention in advance based on expectations about how difficult an upcoming visual task will be. Specifically, they aimed to test whether expecting a more demanding search leads to a narrower focus of attention or instead strengthens attention at the relevant location without changing its spatial extent.

Strengths:

The study addresses a timely and interesting question about how expectations influence the preparation of attention before a task begins. The experimental design is well-suited to isolating anticipatory effects by manipulating expectations about task difficulty independently of moment-to-moment stimulus information. The manuscript is clearly written, and the methods are described in sufficient detail to support transparency and reproducibility.

Weaknesses:

Despite the strengths of the design and the merit of the work, I have a few concerns regarding the analysis and the interpretation of the results.

We thank Reviewer 2 for the positive assessment of the study and for the thoughtful and constructive feedback. The reviewer highlighted several strengths, including the timeliness of the research question, the suitability of the experimental design, and the clarity of the manuscript. In response to the concerns raised, we have: revised the statistical reporting throughout to use Wald z statistics and replaced figure caption t-tests with GLMM-based contrasts; added a caveat in the Discussion acknowledging that the absence of tuning width differences does not definitively rule out changes in attentional scope; and added a paragraph in the Discussion addressing the alternative explanation of general alertness or motivational changes. We address each concern in detail below.

(1) I was somewhat confused by aspects of the behavioural analysis. I may be mistaken, but fixed effects in generalised mixed-effects models are more commonly reported using Wald statistics with beta coefficients rather than F statistics, and the very large degrees of freedom reported here are difficult to interpret. In particular, they appear closer to trial counts than to the number of participants, which raises questions about how statistical uncertainty is being estimated. This concern is compounded by the fact that different statistical approaches appear to yield different conclusions: the generalised mixed-effects models and the pairwise t-tests reported in the figure caption do not fully align. Moreover, the latter are not described in the Methods, and the justification for using them in the figure is not provided. Taken together, this makes it difficult to assess the strength of the behavioural evidence. The reported effects of expectation on behaviour also appear small, and there is no clear cost at uncued locations. This limited behavioural footprint makes it difficult to determine how robust the proposed preparatory mechanism is. It also complicates the interpretation of the neural findings as reflecting a general strategy for optimising task preparation.

We appreciate this observation and agree that reporting Wald statistics is more appropriate for GLMMs. In the revised manuscript, we now report fixed effects as regression coefficients (β), standard errors, z values, and associated p values, rather than Type III F statistics. This reporting more directly reflects the estimation procedure used in

We have also removed the reporting of large denominator degrees of freedom, which reflected the number of trial-level observations but may have been confusing in this context. All models included by-participant random intercepts and random slopes for the within-subject factors, ensuring that statistical uncertainty is appropriately estimated while accounting for the hierarchical structure of the data.

Regarding the pairwise comparisons shown in the figure caption, these previously reflected conventional pairwise t-tests and have now been replaced with contrasts derived from the GLMM using estimated marginal means, consistent with the statistical approach used throughout the manuscript. We have clarified in both the Methods and Results sections that these contrasts are fully model-based and examine the effect of Expectation separately within valid and invalid trials.

Overall, the revised reporting format aligns the statistical presentation more closely with current standards for GLMM analyses and improves interpretability, while leaving the substantive conclusions unchanged.

(2) A central premise of the study is that, if observers proactively narrow their attentional focus when expecting difficult search, this should be reflected in sharper spatial tuning profiles. This prediction is presented as a diagnostic test of whether expectations modulate attentional scope. However, the absence of such sharpening is later taken as evidence that expectations do not alter spatial extent and instead operate exclusively through gain modulation. This inference may be stronger than the data allow. The lack of an observed difference in tuning width does not necessarily rule out changes in attentional scope, particularly if such changes are subtle, temporally limited, or not well captured by the spatial resolution of the approach. As a result, while the findings are consistent with a gain-based account, they do not definitively exclude the possibility that expectations also influence spatial extent, and the logic linking the original prediction to the final conclusion would benefit from a more cautious interpretation.

We thank the reviewer for this important point. We agree that the absence of a tuning width difference does not definitively rule out changes in attentional scope, and we have added a caveat in the Discussion acknowledging that subtle or temporally limited changes may not be fully captured by the spatial resolution of the current approach. We have revised the relevant section to adopt a more cautious interpretation while maintaining that the current findings are most consistent with a gain-based account.

“We note, however, that the absence of a tuning width difference should be interpreted with caution. Subtle or temporally limited changes in attentional scope may not be fully captured by the spatial resolution of the current approach, and we cannot definitively exclude the possibility that expectations also influence spatial extent under some conditions.”

(3) The difference between easy and hard searches in the CTF slope is taken as evidence for enhanced preparatory spatial attention under high expected difficulty. However, these differences could also reflect broader changes in alertness or motivational state between blocks. The behavioural results show a small overall increase in accuracy in expect-hard blocks, which may be consistent with a more general increase in task engagement rather than a spatially specific preparatory mechanism. Although the authors decompose slope differences into amplitude and width parameters, the interpretation still relies on ruling out alternative, more global explanations for enhanced signal strength or reduced variability. This leaves some ambiguity as to whether the observed modulation reflects a specific adjustment of preparatory attention or a more general change in task state.

We thank the reviewer for raising this important alternative explanation. We agree that a general increase in alertness or motivational state could in principle produce broader changes in neural signal strength. We have added a paragraph in the Discussion addressing this concern directly. We highlight two aspects of the data that argue against a purely global account: first, the behavioral benefit of expectation was selective to the cued location with no corresponding effects elsewhere, which is inconsistent with a global alertness account; second, multivariate decoding of expectancy condition remained at chance throughout the cue-target interval, indicating that the two conditions did not produce globally distinct patterns of broadband EEG activity. If general arousal were driving the amplitude differences, we would expect such global pattern differences to be detectable by the classifier. Together, these considerations suggest that the observed modulation reflects spatially specific preparatory gain enhancement rather than a general change in task state. We acknowledge, however, that we cannot fully rule out a contribution of motivational or arousal-related factors, and have added appropriate caveats to the Discussion.

“A related concern is whether the amplitude enhancement observed in expect-hard blocks reflects a spatially specific preparatory mechanism or instead a more general change in alertness or motivational state. Several aspects of the data argue against a purely global account. First, the behavioral benefit of expectation was selective to the cued location, with no corresponding costs or benefits at uncued locations, suggesting that expectancy effects were spatially constrained rather than globally distributed. Second, if expect-hard blocks induced a broadly different neural state through general arousal or motivational engagement, this should manifest as a globally distinct pattern of broadband EEG activity that a multivariate classifier could detect. However, decoding accuracy remained at chance throughout the cue-target interval, indicating that the two expectancy conditions did not produce categorically different spatial patterns of neural activity. Together, these findings suggest that the observed amplitude modulation reflects spatially specific preparatory gain enhancement rather than a global change in task engagement.”
